# Empowerment model construction and empirical study of community-based recruitment of platelet donors

**DOI:** 10.3389/fpubh.2026.1837411

**Published:** 2026-07-01

**Authors:** Danfeng Cheng, Pei Yang, Xu Wan, Yangyaqi Shao, Ying Cheng, Chunyan Yang

**Affiliations:** Chongqing Blood Center, Chongqing, China

**Keywords:** apheresis platelets, community empowerment, public health governance, recruitment strategy, structural equation modeling

## Abstract

**Objective:**

To address the structural mismatch between high clinical demand and limited public participation in platelet donation recruitment in megacities, this study constructed a three-dimensional community empowerment model—organizational empowerment, cognitive empowerment, and service empowerment—based on social capital theory, community governance theory, and planned behavior theory. A cross-sectional questionnaire survey was then conducted to examine statistical associations between key factors and attitudes toward platelet donation via machine collection, as well as blood donation intention, thereby providing empirical evidence for subsequent community recruitment strategy design.

**Methods:**

A multi-stage stratified sampling approach was used to select nine administrative districts and 32 communities in Chongqing according to population size and community type. Permanent residents aged ≥18 years who had lived in the community for at least 6 months were recruited. Of 2,200 distributed questionnaires, 2,004 valid responses were returned (valid response rate: 91.0%). Data were cleaned and analyzed using Excel, SPSS 26.0, and AMOS 26.0. The full sample was used to describe demographic and sociological characteristics, awareness deficits, and recruitment preferences; respondents with prior blood donation history were used to describe blood donation behavior; and 1,964 complete cases were included in confirmatory factor analysis (CFA) and structural equation modeling (SEM). The measurement and structural models were evaluated using Cronbach’s *α*, KMO and Bartlett’s test of sphericity, CFA, average variance extracted (AVE), composite reliability (CR), discriminant validity testing, Pearson correlation analysis, and SEM.

**Results:**

Among respondents, 9.80% reported prior blood donation, and 59.18% of these donors had donated only once in the past year, suggesting limited sustained participation. Misconceptions remained common: 38.47% believed blood donation could harm health, and 31.34% of potential donors identified lengthy procedures as the primary participation barrier. Short science-popularization videos (penetration rate: 79.49%), government-enterprise collaborative recruitment programs (penetration rate: 68.21%), and leadership by Party members and officials in blood donation activities (penetration rate: 71.71%) showed high acceptance. CFA indicated standardized factor loadings of 0.65–0.85; AVE values were > 0.50 and CR values were > 0.70. Model fit was good [CMIN/DF = 1.583 (1–3), RMSEA = 0.017 < 0.05, and NFI, TLI, and CFI all > 0.90]. SEM results showed that institutional trust, fixed donor loyalty, community philanthropic culture, blood usage transparency, and social support norms were all significantly and positively associated with attitudes toward platelet donation via machine collection and blood donation intention.

**Conclusion:**

The community empowerment model developed in this study helps explain variation in residents’ attitudes toward platelet donation via machine collection and blood donation intention from the perspectives of institutional trust, community philanthropic culture, blood usage transparency, and service accessibility. Given the cross-sectional study design, the findings indicate correlations rather than causal effects and do not demonstrate direct improvements in recruitment efficiency attributable to specific recruitment measures. Future studies should incorporate longitudinal follow-up, intervention trials, and cost-effectiveness evaluation to further verify the model’s validity and generalizability in real-world community recruitment practice.

## 1 Introduction

With accelerating urbanization, the mismatch between continuous population growth and medical-resource demand in megacities has become increasingly prominent, and the insufficient supply of mechanically collected platelets has emerged as a critical public health issue requiring urgent attention (1). As an essential blood component for coagulation and hemostasis, a sustained imbalance between platelet supply and demand may reduce emergency response efficiency and weaken outcomes of critical-care treatment. Evidence suggests that megacities—characterized by large populations and concentrated healthcare demand—typically require substantial daily platelet reserves, and this gap often widens further during public health emergencies (2). At present, major Chinese cities face a common challenge in platelet donation recruitment: high clinical demand but low public participation. On the one hand, systematic cognitive bias regarding blood donation persists, and potential donors often misinterpret its health implications (3). On the other hand, fast-paced urban life and complex donation procedures continue to constrain participation. Existing studies have largely focused on single-point approaches, such as digital outreach or enterprise collaboration (4), but a systematic framework grounded in community governance remains limited, making it difficult to fundamentally address the structural mismatch between high demand and low participation in platelet donation recruitment.

To address these challenges, this study establishes a three-dimensional community empowerment framework integrating social capital theory, community governance theory, and planned behavior theory, including organizational empowerment, cognitive empowerment, and service empowerment. Dina Dushkova conceptualizes empowerment as both a process and an outcome, mainly involving (a) individual/cognitive empowerment, (b) development of small mutual-support groups, (c) community organizations, (d) partnership building, and (e) social and political action (5). In the Empower Us project, empowerment is defined as the process through which participants or stakeholders gain decision-making capacity to mobilize resources and institutions toward sustainable development goals, with three key dimensions: (1) access to resources and institutions, (2) mobilization strategies, and (3) willingness to act (6–8). Community empowerment has been widely recognized as a core pathway for promoting grassroots sustainable development and addressing social challenges, receiving sustained attention from governments, non-profit organizations, and academia (9). Prior evidence further indicates that community governance can improve accessibility of public health services (10), while community mobilization can strengthen participation in health-related initiatives (11–13).

In this study, community empowerment is defined as a stakeholder-centered mechanism of mobilization and trust building. Organizational empowerment emphasizes institutional trust and networked mobilization capacity; cognitive empowerment focuses on correcting health misconceptions and strengthening civic responsibility, and is closely related to community philanthropic culture and blood usage transparency; service empowerment aims to reduce participation costs and improve accessibility, thereby supporting blood donation intention and blood donation behavior. This dimensional framework provides a context-specific analytic approach to platelet donation recruitment by integrating individual behavior theory with community empowerment theory and clarifying how external community resources are associated with actual donation-related outcomes.

**Figure 1:**
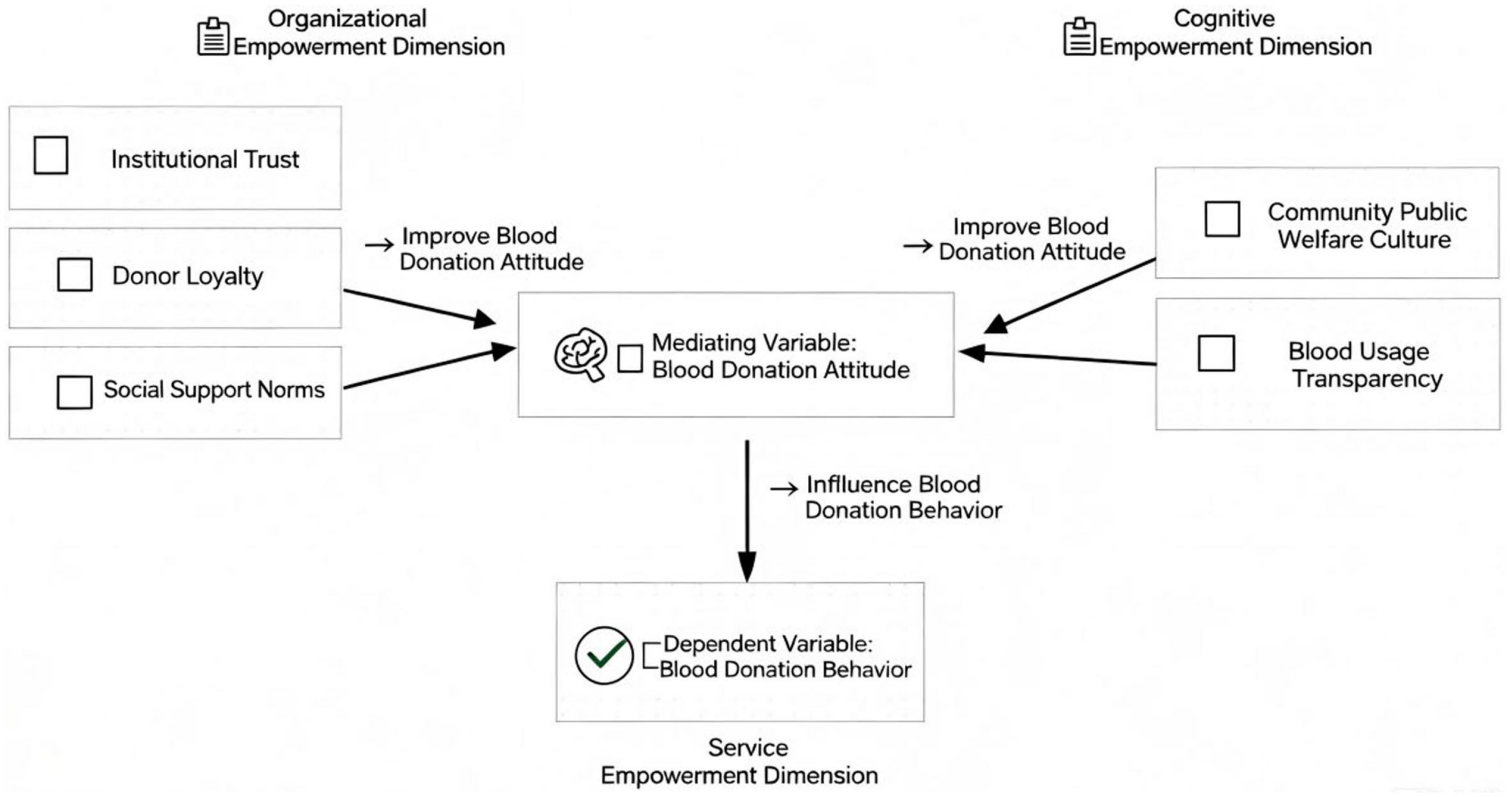
Conceptual structure diagram of variables.

Most previous studies on blood donation have explained willingness primarily through individual knowledge, attitudes, and demographic-sociological characteristics, while relatively less attention has been paid to the combined role of community governance capacity, institutional trust, social norms, and service accessibility in blood donation behavior (14). Whole-blood donation and platelet donation via machine collection differ in both barriers and motivational structure: prosocial motives (e.g., altruism) are frequently reported facilitators, whereas fear-related factors (e.g., syncope, needle phobia) are common barriers. Compared with whole-blood donation, platelet donation via machine collection generally entails stricter eligibility screening and longer collection time, resulting in higher perceived time cost and lower public familiarity with the process (15). Therefore, platelet donation recruitment requires more targeted and systematic recruitment measures. Although existing literature has highlighted the mobilization value of communities and the importance of institutional capacity and governance mechanisms in public health participation (16), these elements have not yet been fully integrated into the specific context of platelet donation recruitment (Figure 1).

Within this framework, the study proposes the following hypotheses: H1: Institutional trust is positively correlated with attitudes toward platelet donation via machine collection for clinical use; H2: Fixed donor loyalty is positively correlated with attitudes toward platelet donation via machine collection for clinical use; H3: Community philanthropic culture is positively correlated with attitudes toward platelet donation via machine collection for clinical use; H4: Blood usage transparency is positively correlated with attitudes toward platelet donation via machine collection for clinical use; H5: Social support norms are positively correlated with attitudes toward platelet donation via machine collection for clinical use; H6: Attitudes toward platelet donation via machine collection for clinical use are positively correlated with blood donation intention; H7: Attitudes toward platelet donation via machine collection for clinical use mediate the relationships between each predictor variable and blood donation intention. This study focuses on community residents in Chongqing and examines both public awareness and prior blood donation behavior to construct and validate a measurable explanatory model of community empowerment. The main innovations are as follows: first, integrating social capital theory, community governance theory, and planned behavior theory into a cross-level framework of “community empowerment → attitude change → behavioral association”; second, focusing on platelet donation via machine collection in megacities, a context characterized by high time costs and strong trust requirements. As this is a cross-sectional survey, causal effects of recruitment measures cannot be inferred and should be further validated through longitudinal or experimental studies.

## 2 Subjects and methods

### 2.1 Study subjects

This study adopted a multi-stage stratified sampling design. In stage 1, nine administrative districts in Chongqing were selected according to population size. In stage 2, 32 communities were selected by community type (old residential neighborhoods, newly developed commercial housing communities, affordable housing communities, and employee residential compounds). In stage 3, survey quotas were allocated according to each community’s resident population, and participants were recruited through community service centers, resident activity sites, and online QR-code access to complete the questionnaire. Inclusion criteria were: (1) age ≥ 18 years; (2) continuous residence in the current community for ≥ 6 months; and (3) ability to understand questionnaire items independently and willingness to participate. Exclusion criteria were severe communication barriers or missing critical questionnaire information. A total of 2,200 questionnaires were distributed, and 2,004 valid questionnaires were collected (effective response rate: 91.0%). The full sample (*n* = 2,004) was used to analyze demographic and sociological characteristics, blood donation-related awareness, recruitment channel preferences, and recognition of community governance measures. Respondents with prior blood donation experience (*n* = 196) were used to describe donation frequency and blood donation behavior. CFA and SEM analyses used complete questionnaire cases (*n* = 1,964); 40 cases with missing key items were excluded from model analysis.

### 2.2 Methods

This study used a self-developed questionnaire. Item development drew on relevant domestic and international scales and was locally adapted to the context of platelet donation via machine collection. Before formal data collection, a pilot survey was conducted in non-target communities (*n* = 150). Based on feedback, culturally adaptive revisions were made to item wording, and three items with factor loadings below 0.50 or with substantial cross-loadings were removed, yielding the final questionnaire. The instrument covered demographic and sociological information, blood donation-related perceptions, participation barriers, acceptance of diversified recruitment methods, evaluation of community governance measures, and items measuring seven latent constructs. Latent variables were rated on a 5-point Likert scale, with higher scores indicating stronger identification or stronger behavioral tendency. The seven constructs included 49 items in total: institutional trust (6 items), fixed donor loyalty (8 items), community philanthropic culture (8 items), blood usage transparency (7 items), social support norms (6 items), attitudes toward platelet donation via machine collection (8 items), and blood donation intention (6 items). Detailed measurement content and theoretical sources are presented in Table A1.

### 2.3 Statistical analysis

#### 2.3.1 Statistical software and Indicator definitions

The dataset was entered, verified, and cleaned in Excel. Descriptive statistics, A one-way ANOVA, reliability analysis, KMO and Bartlett’s tests, and Pearson correlation analyses were conducted using SPSS 26.0. CFA and SEM were performed using AMOS 26.0. All tests were two-tailed, and *p* < 0.05 was considered statistically significant for group differences or path coefficients. Key indicators were defined as follows: response rate refers to the proportion of selections for a specific option among all selections in a multiple-response item, and is used to describe option-selection structure; penetration rate refers to the proportion of respondents selecting a specific option within the total sample (*n* = 2,004), reflecting acceptance and population coverage of a given measure. The reported “42% time reduction” represents respondents’ subjective estimation comparing baseline time (approximately 90 min for conventional platelet donation via machine collection) with optimized expected time, rather than an observed post-intervention effect; therefore, it is used only as a descriptive indicator of service optimization preference and does not imply actual efficiency gains.

#### 2.3.2 Analysis steps

Count data are presented as frequencies and composition percentages (%). According to variable type, analysis of variance was applied across population characteristics to preliminarily examine group differences in blood donation history, awareness deficits, and recruitment preferences. Because the primary aim of this study was to construct a community empowerment model, these between-group comparisons were used mainly to describe sample characteristics and to contextualize the model framework, rather than to support causal inference.

Reliability analysis was conducted for each construct using Cronbach’s *α* to assess internal consistency. All constructs showed Cronbach’s α values above 0.90, and the total questionnaire yielded an overall Cronbach’s α of 0.959. These results support strong item consistency and provide a basis for subsequent CFA and SEM analyses.

Validity analysis included two components: overall suitability testing and construct-level validation. First, the KMO and Bartlett’s tests were used to assess whether the data were appropriate for factor analysis. Second, CFA was conducted for each construct, and AVE and CR were calculated to evaluate convergent validity and construct stability, followed by discriminant validity testing. Construct-level CFA served as the core procedure for validity verification.

Pearson correlation analysis was conducted to examine associations among constructs. Correlation coefficients ranged from 0.340 to 0.453, indicating moderate positive relationships in both direction and strength among latent variables.

SEM was used to test measurement relationships and structural pathways within the proposed theoretical framework. Through latent-variable path modeling, SEM estimates direct and indirect associations while evaluating model-data fit with multiple goodness-of-fit indices and accounting for measurement error more effectively than conventional regression. The prerequisites for SEM application include acceptable reliability and validity of the measurement model, approximate normality of observed data, and significant inter-variable correlations; the present data met these conditions. In AMOS 26.0, the structural model was specified with all variables included for path estimation and mediation testing. To test the mediating role of attitudes toward platelet donation via machine collection, bootstrap resampling (5,000 samples; 95% confidence interval) was performed. A mediation effect was considered significant when the 95% confidence interval did not include 0.

#### 2.3.3 Correspondence between theoretical and empirical variables

To align theoretical interpretation with empirical testing, this study mapped the three-dimensional empowerment framework onto SEM constructs. Organizational empowerment corresponded to institutional trust, fixed donor loyalty, and social support norms, emphasizing the formation of sustainable mobilization networks through community Party organizations to strengthen resident trust and participation norms. Cognitive empowerment corresponded to community philanthropic culture and blood usage transparency, focusing on correcting misconceptions about blood donation through health communication and improving understanding of the social value of platelet donation. Service empowerment referred to enabling conditions that facilitate the translation of donation attitudes into blood donation behavior, including convenient appointment and participation arrangements.

### 2.4 Medical ethics

This study was approved by the Ethical Review Committee of Chongqing Blood Center (Approval No. L20250045). The protocol complied with the Declaration of Helsinki and relevant domestic regulations governing medical research involving human participants. All participants provided written informed consent before enrollment. Prior to questionnaire administration, trained staff explained the study objectives, questionnaire content, and data confidentiality procedures in detail. Participation was fully voluntary based on informed understanding, and no exemption from informed consent was applied. Questionnaire data were anonymized by removing personal identifiers (e.g., names and contact information), and all data were used solely for statistical analysis in this study without external disclosure.

## 3 Results

### 3.1 Demographic and sociological characteristics of survey participants (*n* = 2,004)

A total of 2,004 respondents were included. Age was concentrated in the 26–35-year group (34.28%), followed by the 18–25-year group (26.85%). Gender distribution was relatively balanced, with 975 males (48.65%) and 1,029 females (51.34%). Occupation was mainly corporate employees (34.33%) and students (26.84%). Regarding residence context, newly developed commercial housing communities accounted for the largest proportion (54.49%). Educational attainment was predominantly bachelor’s degree or above (58.13%). Married participants represented 45.30% of the sample (Table 1).

**Table 1:** Age and gender distribution of survey participants (*n* = 2,004).

Name	Option	Frequency	Percentage %
Sex	Man	975	48.65
Your gender?	Woman	1,029	51.34
Age	18–25 years old	538	26.85
Which age range does your age fall into?	Ages 26–35	687	34.28
Which age range does your age fall into?	36–45 years of age	450	22.45
Which age range does your age fall into?	46–55 years of age	191	9.53
Which age range does your age fall into?	55 years of age or older	138	6.88
Occupation	Civil servants/staff of public institutions	166	8.28
What is your profession?	Enterprise employees	688	34.33
What is your profession?	Freelancer	409	20.40
What is your profession?	Student	538	26.84
What is your profession?	Retiree	39	1.94
What is your profession?	Other	164	8.18
Community type	Old residential area	560	27.94
What type of community are you in?	Newly constructed residential community	1,092	54.49
What type of community are you in?	Affordable housing communities	219	10.92
What type of community are you in?	Family compound of the unit	133	6.63
How long have you been living in your current community?	Within 1 year	333	16.61
How long have you lived in your current community?	1–3 years	637	31.78
How long have you lived in your current community?	3–5 years	490	24.45
How long have you lived in your current community?	More than 5 years	544	27.14
highest education	Middle school and below	161	8.03
What is your highest educational qualification?	High school/secondary vocational school	206	10.27
What is your highest educational qualification?	Junior college	472	23.55
What is your highest educational qualification?	Undergraduate course	795	39.67
What is your highest educational qualification?	Master’s degree or higher	370	18.46
Marital status	Unmarried	801	39.97
What is your marital status?	Married	908	45.30
What is your marital status?	Dissociation	212	10.57
What is your marital status?	Bereft of one’s spouse	83	4.14

### 3.2 Current status of blood donation among survey participants

Among all 2,004 respondents, 196 reported prior blood donation history (9.80%), while 1,808 did not (90.20%). The analysis in this subsection focuses on the 196 respondents with donation experience. Results showed that 59.18% had donated only once, whereas frequent donors (≥4 donations) accounted for only 7.14%, indicating low donation frequency and limited sustained participation in blood donation behavior within this sample (Table 2).

**Table 2:** Current blood donation status of survey participants.

Metric	Option	Frequency	Percentage %
Has any experience with blood donation	Have	196	9.80%
	Not have	1808	90.20%
Number of blood donations in the past year (*n* = 196)	1 time	116	59.18%
	2 times	46	23.40%
	3 times	20	10.20%
	4 times or more	14	7.14%
Total number of blood donations (*n* = 196)	1–5 times	114	58.16%
	6–10 times	46	23.46%
	11–15 times	15	7.65%
	16–20 times	15	7.65%
	20 or more times	6	3.06%

### 3.3 Participation in community blood donation activities and satisfaction with leadership engagement (*n* = 2,004)

Among the 2,004 respondents, 64.97% reported never participating in community-organized blood donation publicity activities, whereas 35.03% had participated. Among the 688 participants in such activities, 69.91% reported being “very satisfied” or “somewhat satisfied” with leadership engagement by community officials in blood donation promotion, while 12.96% reported clear dissatisfaction. These findings suggest that community-level publicity and organizational guidance for blood donation can still be strengthened (Table 3).

**Table 3:** Participation rate in community blood donation activities and satisfaction with leadership by officials.

Index classification	Participation status/satisfaction level	Frequency	Percentage %
Participation rate in community blood donation activities	Deny	1,276	64.97%
	Yes	688	35.03%
Satisfaction with the leading role of community officials (based on feedback from only 688 participants):	Fairly satisfied	281	40.84%
	Very satisfied	200	29.07%
	Same as	118	17.13%
	Not very satisfied	60	8.71%
	Very dissatisfied	29	4.25%

### 3.4 Public awareness deficiencies regarding donations (*n* = 2,004)

Analysis of awareness deficits regarding platelet donation via machine collection showed that 38.47% of respondents believed donation would harm health, representing the most common misconception. In addition, 25.70% were unclear about the clinical recipients of platelets, and 19.16% did not recognize that component blood donation generally does not cause health damage. Overall, public understanding of health effects, clinical use, and safety of platelet donation via machine collection remains insufficient (Table 4).

**Table 4:** Analysis of public perceptual disorders (*n* = 2,004).

Type of cognitive resistance	Frequency	Percentage %	Original ratio
Misunderstandings about health relationships	771	38.47%	38.47%
Un明确了 target person	515	25.70%	25.70%
Not aware of any harm	384	19.16%	19.16%
The process is cumbersome and complex	206	10.28%	10.28%
Fear and unfounded rumors	128	6.39%	6.39%

### 3.5 Restrictive factors affecting donation participation (*n* = 2,004)

Among individual factors affecting participation in platelet donation via machine collection, 31.34% of respondents identified “the prolonged duration of platelet donation via machine collection” as the primary barrier, 25.60% identified fear of blood-draw pain, and 19.21% reported concern about overall time commitment. Taken together, perceived time cost and anticipated procedural discomfort were the main individual-level constraints on participation (Table 5).

**Table 5:** Statistical results of individual factors limiting participation of potential platelet donors (*n* = 2,004).

Limiting factor	Frequency	Percentage %	Original ratio	Key findings
The platelet collection process for blood donation takes too long.	628	31.34%	31.34%	The most critical obstacle: over 30% of respondents expressed concern.
Fear of pain, fear of the blood collection procedure	513	25.60%	25.60%	The second major obstacle is the primary psychological barrier.
Concerned about potential impacts on daily work and routine life rhythms	385	19.21%	19.21%	The time coordination issue is significant.
Lack of confidence in the techniques for collecting component blood	219	10.93%	10.93%	Insufficient trust in technology
My overall health condition is not good.	162	8.08%	8.08%	Health condition limitations
Other factor	97	4.84%	4.84%	Unclear factors

### 3.6 Feasibility of multi-channel recruitment methods (multiple-response analysis, *n* = 2,004)

The feasibility of multi-channel recruitment methods was evaluated using both response rate and penetration rate. Short science-communication videos showed the highest penetration rate (79.49%), followed by enterprise collaboration that integrates blood donation into employee benefits or team-building arrangements (68.21%), and online registration with simplified procedures (56.44%). These results suggest high acceptance of recruitment measures based on digital dissemination, organizational collaboration, and process optimization. Here, response rate reflects selection frequency across options, while penetration rate reflects population-level acceptance coverage; together they characterize multiple-response results from complementary perspectives (Table 6).

**Table 6:** Summary of feasible multi-channel recruitment methods for component blood donation (*n* = 2,004).

Question type	Option	Response count (n)	Response ratio (%)	Popularity rate (%)
The feasibility of using multiple recruitment methods	Use short video platforms to create engaging educational videos on blood donation.	1,593	26.44	79.49
	Work with companies to include blood donation as part of employee benefits or team-building programs.	1,367	22.69	68.21
	Implement online blood donation registration and streamline the process	1,131	18.78	56.44
	In collaboration with university student organizations, we organize themed blood donation events on campus.	617	10.24	30.79
	Establish a reward mechanism for donor recommendations to encourage existing donors to recruit new ones.	369	6.13	18.41
	Hold a blood donation culture festival to promote and publicize blood donation efforts extensively.	443	7.35	22.11
	Set up mobile blood donation vehicles and promotional stalls in large shopping malls.	337	5.59	16.82
	Other	167	2.77	8.33
Subtotal	Total responses	6,024	100	-

### 3.7 Feasibility of community governance recruitment measures (multiple-response analysis, *n* = 2,004)

From the perspective of community governance measures, “organizing Party members and officials to lead blood donation activities” reached a penetration rate of 71.71%, and “providing free health check-ups and consultation services” reached 60.58%, both ranking highest and indicating strong practical feasibility. Compared with other recruitment measures, these two approaches were more readily accepted by residents and may be prioritized in community-based platelet donation recruitment practice (Table 7).

**Table 7:** Blood donor recruitment measures at the community governance level (*n* = 2,004).

Question type	Option	Response count (n)	Response ratio (%)	Popularity rate (%)
Community governance measures	Organize party members and officials within the community to take the lead in donating blood.	1,437	24.02	71.71
	Provide free medical examinations or health consultations for blood donors	1,214	20.29	60.58
	Establish a dedicated community blood donation fund to reward donors.	841	14.06	41.97
	We collaborate with nearby schools to promote science education among teenagers.	691	11.55	34.48
	Establish a community-based donor mutual assistance network	579	9.68	28.89
	Conduct a blood donation knowledge competition and reward the winners	554	9.26	27.65
	Use community radio to conduct regular publicity campaigns.	487	8.14	24.3
	Other	179	2.99	8.93
Subtotal	Total responses	5,982	100	-

### 3.8 Results of item validity and reliability tests

#### 3.8.1 Reliability by dimension

Reliability testing showed that Cronbach’s *α* coefficients for all seven latent constructs were above 0.90, indicating strong internal consistency across dimensions. The overall Cronbach’s α of the full questionnaire was 0.959, further demonstrating high scale stability and consistency. These results satisfy the reliability requirements for subsequent correlation and SEM analyses (Table 8).

**Table 8:** Reliability statistics for each variable.

Reliability statistics		
Dimension name	Cronbach alpha	Number of terms
institutional trust	0.903	6
Enhancing the loyalty of regular blood donors	0.929	8
Community public welfare culture	0.939	8
Blood transparency	0.920	7
Social support norms	0.912	6
Attitudes toward donating platelets collected by machine	0.935	8
Intention to donate blood	0.928	6
Overall	0.959	49

#### 3.8.2 Validity results

Validity testing showed an overall KMO value of 0.973, and Bartlett’s test yielded χ^2^ = 67,274.641 (*p* < 0.001), indicating that the dataset was appropriate for factor analysis. Overall, the scale demonstrated satisfactory construct validity and supported further structural relationship testing (Table 9).

**Table 9:** KMO and Bartlett’s test.

KMO sample appropriateness measure.		0.973
Bartlett sphericity test	Approximate chi-square	67274.641
	Freedom degree	1176.000
	conspicuousness	0.000

#### 3.8.3 Variance explanatory rate analysis

Based on factor extraction and information-content assessment, seven factors with eigenvalues greater than 1 were retained. After rotation, the variance explanatory rates were 11.556, 11.296, 11.008, 9.587, 8.863, 8.399, and 8.360%, respectively, and the cumulative rotated variance explanatory rate reached 69.068% (Table 10). This exceeded the commonly accepted 60% threshold for factor analysis, indicating adequate information coverage by the extracted factors.

**Table 10:** Variance explanatory rate.

Ingredient	Initial eigenvalue			Extract the sum of square values of the loads			Sum of squares of rotational loads		
	Total	Variance percentage	Accumulate %	Total	Variance percentage	Accumulate %	Total	Variance percentage	Accumulate %
1	16.616	33.910	33.910	16.616	33.910	33.910	5.662	11.556	11.556
2	3.421	6.983	40.893	3.421	6.983	40.893	5.535	11.296	22.851
3	3.102	6.330	47.223	3.102	6.330	47.223	5.394	11.008	33.859
4	2.847	5.810	53.033	2.847	5.810	53.033	4.698	9.587	43.446
5	2.810	5.735	58.768	2.810	5.735	58.768	4.343	8.863	52.309
6	2.635	5.377	64.145	2.635	5.377	64.145	4.116	8.399	60.708
7	2.412	4.923	69.068	2.412	4.923	69.068	4.096	8.360	69.068
8	0.739	1.508	70.576						
9	0.461	0.940	71.516						
10	0.450	0.918	72.434						
11	0.441	0.901	73.335						
12	0.437	0.893	74.227						
13	0.432	0.882	75.109						
14	0.420	0.857	75.966						
15	0.416	0.848	76.815						
16	0.411	0.839	77.654						
17	0.404	0.824	78.477						
18	0.402	0.820	79.297						
19	0.397	0.810	80.107						
20	0.389	0.794	80.901						
21	0.386	0.788	81.690						
22	0.385	0.785	82.475						
23	0.376	0.767	83.242						
24	0.374	0.764	84.006						
25	0.370	0.756	84.761						
26	0.356	0.727	85.489						
27	0.353	0.721	86.210						
28	0.352	0.718	86.928						
29	0.349	0.712	87.640						
30	0.345	0.704	88.344						
31	0.339	0.692	89.036						
32	0.334	0.682	89.718						
33	0.330	0.674	90.392						
34	0.326	0.665	91.057						
35	0.318	0.649	91.705						
36	0.317	0.647	92.352						
37	0.312	0.636	92.988						
38	0.310	0.632	93.620						
39	0.305	0.623	94.243						
40	0.304	0.621	94.864						
41	0.300	0.613	95.477						
42	0.295	0.601	96.078						
43	0.291	0.594	96.673						
44	0.286	0.584	97.257						
45	0.281	0.574	97.831						
46	0.276	0.562	98.393						
47	0.267	0.546	98.939						
48	0.265	0.541	99.480						
49	0.255	0.520	100.000						

#### 3.8.4 Confirmatory factor analysis

As shown in Table 11, CMIN/DF = 1.583 (acceptable range: 1–3), RMSEA = 0.017 (< 0.05), and all fit indices (NFI, TLI, CFI) were above 0.90. These results indicate excellent fit of the CFA model.

**Table 11:** Fit test for the CFA model of the scale.

Metric	Reference standard	Actual measurement results
CMIN/DF	1–3: excellent; 3–5: good	1.583
RMSEA	<0.05 indicates excellent; <0.08 indicates good	0.017
NFI	>0.9 indicates excellent;>0.8 indicates good	0.974
TLI	>0.9 indicates excellent;>0.8 indicates good	0.99
CFI	>0.9 indicates excellent;>0.8 indicates good	0.959

#### 3.8.5 Results of standardized factor loadings and model AVE/CR indicators

As shown in Table 12, standardized factor loadings for all items were greater than 0.60 in absolute value and were statistically significant, supporting good measurement performance. In addition, AVE values for all dimensions exceeded 0.50 and CR values exceeded 0.80, indicating robust convergent validity and composite reliability across constructs.

**Table 12:** Results of standardized factor loadings and model AVE/CR indicators.

Path relationship			Standard load factor	AVE	CR
A1	<−--	Institutional trust	0.784	0.607	0.903
A2	<−--	Institutional trust	0.779		
A3	<−--	Institutional trust	0.768		
A4	<−--	Institutional trust	0.781		
A5	<−--	Institutional trust	0.783		
A6	<−--	Institutional trust	0.781		
B1	<−--	Enhancing the loyalty of regular blood donors	0.784	0.62	0.929
B2	<−--	Enhancing the loyalty of regular blood donors	0.794		
B3	<−--	Enhancing the loyalty of regular blood donors	0.793		
B4	<−--	Enhancing the loyalty of regular blood donors	0.781		
B5	<−--	Enhancing the loyalty of regular blood donors	0.796		
B6	<−--	Enhancing the loyalty of regular blood donors	0.790		
B7	<−--	Enhancing the loyalty of regular blood donors	0.789		
B8	<−--	Enhancing the loyalty of regular blood donors	0.771		
C1	<−--	Community public welfare culture	0.819	0.657	0.939
C2	<−--	Community public welfare culture	0.817		
C3	<−--	Community public welfare culture	0.806		
C4	<−--	Community public welfare culture	0.806		
C5	<−--	Community public welfare culture	0.812		
C6	<−--	Community public welfare culture	0.801		
C7	<−--	Community public welfare culture	0.806		
C8	<−--	Community public welfare culture	0.815		
D1	<−--	Blood transparency	0.797	0.623	0.92
D2	<−--	Blood transparency	0.782		
D3	<−--	Blood transparency	0.788		
D4	<−--	Blood transparency	0.787		
D5	<−--	Blood transparency	0.795		
D6	<−--	Blood transparency	0.776		
D7	<−--	Blood transparency	0.801		
E1	<−--	Social support norms	0.788	0.632	0.912
E2	<−--	Social support norms	0.817		
E3	<−--	Social support norms	0.786		
E4	<−--	Social support norms	0.798		
E5	<−--	Social support norms	0.811		
E6	<−--	Social support norms	0.770		
F1	<−--	Attitudes toward donating platelets collected by machine	0.804	0.644	0.935
F2	<−--	Attitudes toward donating platelets collected by machine	0.795		
F3	<−--	Attitudes toward donating platelets collected by machine	0.802		
F4	<−--	Attitudes toward donating platelets collected by machine	0.805		
F5	<−--	Attitudes toward donating platelets collected by machine	0.791		
F6	<−--	Attitudes toward donating platelets collected by machine	0.801		
F7	<−--	Attitudes toward donating platelets collected by machine	0.812		
F8	<−--	Attitudes toward donating platelets collected by machine	0.807		
G1	<−--	Intention to donate blood	0.828	0.683	0.928
G2	<−--	Intention to donate blood	0.817		
G3	<−--	Intention to donate blood	0.839		
G4	<−--	Intention to donate blood	0.815		
G5	<−--	Intention to donate blood	0.832		
G6	<−--	Intention to donate blood	0.826		

#### 3.8.6 Discriminant validity

As shown in Table 13, for every pair of constructs, the inter-construct correlation coefficient was lower than the square root of the corresponding AVE, indicating satisfactory discriminant validity among all dimensions.

**Table 13:** Discriminant validity: correlation coefficients and square roots of AVE.

Dimension name	Institutional trust	Enhancing the loyalty of regular blood donors	Community public welfare culture	Blood transparency	Social support norms	Attitudes toward donating platelets collected by machine	Intention to donate blood
Institutional trust	**0.779**						
Enhancing the loyalty of regular blood donors	0.398	**0.787**					
Community public welfare culture	0.377	0.421	**0.810**				
Blood transparency	0.393	0.486	0.454	**0.789**			
Social support norms	0.378	0.474	0.412	0.494	**0.795**		
Attitudes toward donating platelets collected by machine	0.399	0.467	0.437	0.481	0.449	**0.802**	
Intention to donate blood	0.372	0.450	0.429	0.463	0.442	0.436	**0.826**

The diagonal numbers represent the square root of AVE. The bold values indicates AVE values exceed 0.5, indicating that the analytical data demonstrate strong convergent validity.

### 3.9 Pearson correlation analysis (*n* = 1,964)

Pearson correlation analysis showed that institutional trust, fixed donor loyalty, community philanthropic culture, blood usage transparency, social support norms, attitudes toward platelet donation via machine collection, and blood donation intention were all significantly and positively correlated with one another (all *p* < 0.001). Correlation coefficients ranged from 0.340 to 0.453, indicating moderate positive associations among variables (Table 14).

**Table 14:** Pearson correlation analysis.

Dimension name	1	2	3	5	6	7	8
Institutional trust	1						
Enhancing the loyalty of regular blood donors	0.365***	1					
Community public welfare culture	0.347***	0.394***	1				
Blood transparency	0.358***	0.450***	0.422***	1			
Social support norms	0.343***	0.436***	0.381***	0.453***	1		
Attitudes toward donating platelets collected by machine	0.367***	0.435***	0.409***	0.446***	0.415***	1	
Intention to donate blood	0.340***	0.419***	0.401***	0.428***	0.407***	0.407***	1

*p < 0.05, **p < 0.01, ***p < 0.001.

### 3.10 Structural equation modeling

SEM was applied to test the measurement relationships and structural associations within the complex theoretical framework. By modeling latent-variable pathways, SEM estimates both direct and indirect effects while providing an integrated assessment of model-data fit through indices such as CMIN/DF, CFI, and RMSEA. Relative to traditional regression approaches, this method offers better handling of measurement error and more stable parameter estimation.

#### 3.10.1 SEM model fit test

As shown in Table 15, CMIN/DF = 1.583 (acceptable range: 1–3), RMSEA = 0.017 (< 0.05), and all fit indices (NFI, TLI, CFI) exceeded 0.90. These results indicate excellent fit of the SEM model for blood donation intention.

**Table 15:** Fit test for the CFA model of the scale.

Metric	Reference standard	ACTUAL measurement results
CMIN/DF	1–3: Excellent; 3–5: Good	1.583
RMSEA	<0.05 indicates excellent; <0.08 indicates good	0.017
NFI	>0.9 indicates excellent;>0.8 indicates good	0.974
TLI	>0.9 indicates excellent;>0.8 indicates good	0.99
CFI	>0.9 indicates excellent;>0.8 indicates good	0.99

#### 3.10.2 Path analysis and hypothesis testing

Given satisfactory model fit, structural path analysis showed that institutional trust (*β* = 0.134, C. R. = 5.600, *p* < 0.001), fixed donor loyalty (*β* = 0.180, C. R. = 7.016, *p* < 0.001), community philanthropic culture (*β* = 0.160, C. R. = 6.597, *p* < 0.001), blood usage transparency (*β* = 0.192, C. R. = 7.269, *p* < 0.001), and social support norms (*β* = 0.152, C. R. = 5.905, *p* < 0.001) were all significantly and positively associated with attitudes toward platelet donation via machine collection. Institutional trust (*β* = 0.091, C. R. = 3.742, *p* < 0.001), fixed donor loyalty (*β* = 0.149, C. R. = 5.649, *p* < 0.001), community philanthropic culture (*β* = 0.147, C. R. = 5.897, *p* < 0.001), blood usage transparency (*β* = 0.157, C. R. = 5.777, *p* < 0.001), social support norms (*β* = 0.143, C. R. = 5.411, *p* < 0.001), and attitudes toward platelet donation via machine collection (β = 0.127, C. R. = 4.892, p < 0.001) were also significantly and positively associated with blood donation intention. Therefore, H1–H6 were supported (Table 16).

**Table 16:** Path indicators.

Way			Non-standard path coefficient	S. E.	C. R.	*P*	Standard path coefficient
Attitudes toward donating platelets collected by machine	<−-	Institutional trust	0.136	0.024	5.600	0.000***	0.134
Attitudes toward donating platelets collected by machine	<−-	Enhancing the loyalty of regular blood donors	0.187	0.027	7.016	0.000***	0.180
Attitudes toward donating platelets collected by machine	<−-	Community public welfare culture	0.152	0.023	6.597	0.000***	0.160
Attitudes toward donating platelets collected by machine	<−-	Blood transparency	0.193	0.027	7.269	0.000***	0.192
Attitudes toward donating platelets collected by machine	<−-	Social support norms	0.159	0.027	5.905	0.000***	0.152
Intention to donate blood	<−-	Institutional trust	0.098	0.026	3.742	0.000***	0.091
Intention to donate blood	<−-	Enhancing the loyalty of regular blood donors	0.164	0.029	5.649	0.000***	0.149
Intention to donate blood	<−-	Community public welfare culture	0.148	0.025	5.897	0.000***	0.147
Intention to donate blood	<−-	Blood transparency	0.167	0.029	5.777	0.000***	0.157
Intention to donate blood	<−-	Social support norms	0.158	0.029	5.411	0.000***	0.143
Intention to donate blood	<−-	Attitudes toward donating platelets collected by machine	0.135	0.028	4.892	0.000***	0.127

*p < 0.05, **p < 0.01, ***p < 0.001.

#### 3.10.3 Effect test results

Attitudes toward platelet donation via machine collection showed partial mediation between institutional trust, fixed donor loyalty, community philanthropic culture, blood usage transparency, social support norms, and blood donation intention. The standardized indirect effects were 0.017, 0.023, 0.020, 0.024, and 0.019, respectively, and the corresponding 95% bootstrap confidence intervals did not include 0, indicating statistically significant indirect pathways. Because direct effects from antecedent variables to blood donation intention remained significant, the mediation was partial rather than full. These findings support H7 and suggest that improving donation attitudes may be a key link in the associations between institutional trust, community philanthropic culture, blood usage transparency, social support norms, and behavioral tendency (Table 17).

**Table 17:** Effect test.

Parameter	Estimate	Lower	Upper	Standard estimate	Standard lower	Standard Upper	Hypothesis
Blood donation intention <-Attitude toward platelet donation <-Institutional trust level (indirect effect)	0.018	0.01	0.032	0.017	0.009	0.028	Some intermediaries
Blood donation behavior intention <-Institutional trust level (direct effect)	0.098	0.046	0.151	0.091	0.043	0.141	Found
Blood donation behavior intention <-Attitude toward platelet donation <−Loyalty of regular donors (indirect effect)	0.025	0.013	0.041	0.023	0.013	0.038	Some intermediaries
Blood donation behavior intention <-Fixed donor loyalty (direct effect)	0.164	0.104	0.227	0.149	0.095	0.202	Found
Blood donation behavior intention <-Attitude toward platelet donation <-Community public welfare culture (indirect effect)	0.02	0.011	0.033	0.02	0.011	0.033	Some intermediaries
Blood donation behavior intention <-Community public welfare culture (direct effect)	0.148	0.092	0.201	0.147	0.093	0.2	Found
Blood donation intention <-Attitude toward platelet donation <−Transparency of blood usage (indirect effect)	0.026	0.014	0.043	0.024	0.013	0.04	Some intermediaries
Blood donation behavior intention <−Transparency of blood usage (direct effect)	0.167	0.096	0.227	0.157	0.092	0.212	Found
Blood donation behavior intention <-Attitude toward platelet donation <-Social support norms (indirect effect)	0.021	0.012	0.036	0.019	0.011	0.032	Some intermediaries
Blood donation behavior intention <-Social support norms (direct effect)	0.158	0.094	0.226	0.143	0.085	0.202	Found

Lower indicates the lower bound of the 95% confidence interval, and Upper indicates the upper bound. A confidence interval that does not include 0 indicates a significant effect.

### 3.11 Difference analysis

An independent-samples *t*-test was used to examine gender differences in institutional trust, fixed donor loyalty, community philanthropic culture, blood usage transparency, social support norms, attitudes toward platelet donation via machine collection, and blood donation intention. As shown in Table 18, no significant gender differences were found for any dimension (*p* > 0.05).

**Table 18:** Results of gender *t*-test analysis (*n* = 1,964).

Dimension name	Your gender (Average ± SD)		*t*	*p*
	Men (*n* = 958)	Women (*n* = 1,006)		
Institutional trust	3.280 ± 1.000	3.283 ± 0.964	−0.073	0.942
Enhancing the loyalty of regular blood donors	3.384 ± 0.979	3.344 ± 0.960	0.910	0.363
Community public welfare culture	3.444 ± 1.019	3.473 ± 1.007	−0.646	0.518
Blood transparency	3.438 ± 0.965	3.396 ± 0.978	0.963	0.336
Social support norms	3.351 ± 0.988	3.402 ± 0.972	−1.157	0.247
Attitudes toward donating platelets collected by machine	3.388 ± 0.976	3.348 ± 0.985	0.909	0.363
Intention to donate blood	3.371 ± 1.057	3.395 ± 1.047	−0.514	0.608

A one-way ANOVA was performed to test age-group differences in institutional trust, fixed donor loyalty, community philanthropic culture, blood usage transparency, social support norms, attitudes toward platelet donation via machine collection, and blood donation intention. As shown in Table 19, none of these indicators differed significantly across age groups (*p* > 0.05), suggesting limited age-related variation in these dimensions.

**Table 19:** Results of age ANOVA (*n* = 1,964).

Dimension name	Which age range does your age fall into? (Average ± SD)					*F*	*p*
	18–25 years old (*n* = 526)	Ages 26–35 (*n* = 673)	36–45 years of age (*n* = 443)	46–55 years of age (*n* = 186)	55 years of age or older (*n* = 136)		
Institutional trust	3.260 ± 0.979	3.310 ± 0.988	3.264 ± 0.986	3.270 ± 0.979	3.294 ± 0.954	0.255	0.907
Enhancing the loyalty of regular blood donors	3.339 ± 0.985	3.386 ± 0.955	3.397 ± 0.958	3.358 ± 0.948	3.242 ± 1.041	0.841	0.499
Community public welfare culture	3.406 ± 1.023	3.500 ± 1.018	3.470 ± 1.014	3.511 ± 0.974	3.351 ± 0.989	1.151	0.331
Blood transparency	3.396 ± 0.938	3.466 ± 0.977	3.356 ± 1.021	3.356 ± 0.951	3.535 ± 0.925	1.602	0.171
Social support norms	3.330 ± 0.982	3.456 ± 0.985	3.350 ± 0.941	3.349 ± 1.027	3.289 ± 0.998	1.784	0.129
Attitudes toward donating platelets collected by machine	3.382 ± 0.966	3.406 ± 0.993	3.358 ± 0.962	3.273 ± 1.013	3.278 ± 0.992	1.010	0.401
Intention to donate blood	3.343 ± 1.065	3.411 ± 1.043	3.382 ± 1.018	3.407 ± 1.101	3.376 ± 1.090	0.335	0.854

A one-way ANOVA examined differences across community types in seven dimensions, including institutional trust (Table 20). No significant differences were observed for four dimensions (e.g., fixed donor loyalty; *p* > 0.05). However, significant between-community-type differences were observed for institutional trust, community philanthropic culture, and social support norms (*p* < 0.05). Specifically, institutional trust differed significantly at the 0.05 level (*F* = 2.859, *p* = 0.036), with mean scores ranked as employee residential compounds > affordable housing communities > newly developed commercial housing communities > old residential neighborhoods. Similar significant differences were observed for community philanthropic culture (*F* = 2.893, *p* = 0.034) and social support norms (*F* = 3.622, *p* = 0.013).

**Table 20:** Results of ANOVA for community types (*n* = 1,964).

Dimension name	What type of community are you in? (Average ± SD)				*F*	*p*
	Old residential area (*n* = 548)	Newly constructed residential community (*n* = 1,071)	Affordable housing communities (*n* = 216)	Family compound of the unit (*n* = 129)		
Institutional trust	3.248 ± 1.019	3.269 ± 0.960	3.281 ± 0.999	3.522 ± 0.943	2.859	0.036*
Enhancing the loyalty of regular blood donors	3.313 ± 0.995	3.386 ± 0.938	3.349 ± 1.008	3.414 ± 1.044	0.820	0.483
Community public welfare culture	3.471 ± 1.031	3.421 ± 1.009	3.475 ± 0.966	3.695 ± 1.014	2.893	0.034*
Blood transparency	3.464 ± 0.968	3.383 ± 0.962	3.403 ± 1.005	3.516 ± 1.004	1.317	0.267
Social support norms	3.386 ± 0.961	3.334 ± 0.991	3.414 ± 0.965	3.628 ± 0.962	3.622	0.013*
Attitudes toward donating platelets collected by machine	3.339 ± 0.986	3.353 ± 0.974	3.447 ± 0.961	3.474 ± 1.042	1.205	0.307
Intention to donate blood	3.364 ± 1.042	3.354 ± 1.061	3.472 ± 1.037	3.554 ± 1.030	1.983	0.114

*p < 0.05, **p < 0.01, **p < 0.001.

A one-way ANOVA was used to compare institutional trust, fixed donor loyalty, and related indicators across different durations of residence in the current community (Table 21). No significant between-group differences were found for fixed donor loyalty (*p* > 0.05), whereas institutional trust differed significantly (*p* < 0.05). Specifically, residence duration was significantly associated with institutional trust at the 0.05 level (*F* = 3.390, *p* = 0.017), and mean scores were ranked as 1–3 years > 3–5 years > within 1 year > over 5 years.

**Table 21:** Results of ANOVA by duration of residence in the current community (*n* = 1,964).

Dimension name	How long have you lived in your current community? (Average ± SD)				*F*	*p*
	Within 1 year (*n* = 324)	1–3 years (*n* = 625)	3–5 years (*n* = 482)	More than 5 years (*n* = 533)		
Institutional trust	3.239 ± 0.997	3.347 ± 0.965	3.334 ± 0.997	3.183 ± 0.969	3.390	0.017*
Enhancing the loyalty of regular blood donors	3.380 ± 0.993	3.369 ± 0.971	3.434 ± 0.939	3.283 ± 0.976	2.099	0.098
Community public welfare culture	3.477 ± 1.029	3.490 ± 1.020	3.467 ± 0.995	3.404 ± 1.011	0.759	0.517
Blood transparency	3.447 ± 0.936	3.452 ± 0.983	3.438 ± 0.972	3.337 ± 0.977	1.654	0.175
Social support norms	3.388 ± 1.010	3.419 ± 0.987	3.424 ± 0.932	3.279 ± 0.992	2.549	0.054
Attitudes toward donating platelets collected by machine	3.390 ± 0.966	3.423 ± 0.972	3.357 ± 0.975	3.297 ± 1.004	1.656	0.174
Intention to donate blood	3.403 ± 1.043	3.401 ± 1.052	3.398 ± 1.032	3.336 ± 1.075	0.484	0.694

## 4 Discussion

This study addressed low participation rates, limited sustainability, and high time costs in platelet donation recruitment in megacities by developing and testing a three-dimensional community empowerment model that includes organizational empowerment, cognitive empowerment, and service empowerment. The findings showed that institutional trust, loyalty among regular blood donors, community philanthropic culture, transparency in blood usage, and social support norms were all significantly and positively associated with attitudes toward platelet donation, and these attitudes were further positively associated with blood donation behavior. These results suggest that organizational trust, community philanthropic culture, and transparent governance at the community level may influence residents’ acceptance of platelet donation; however, because of the cross-sectional design, these pathways should not be interpreted as confirmed causal mechanisms.

From the perspective of sample composition, individuals aged 25–35 accounted for 34.28%, while those aged 18–25 accounted for 26.85%, indicating that younger adults constitute the core population for community platelet donation recruitment. Previous studies have shown that platelet donation via machine collection places relatively high demands on cardiovascular function and platelet regenerative capacity, and young adults generally exhibit physiological advantages in these domains (17–20). Female participation reached 51.35%, slightly higher than that of males; this pattern may reflect both the role of community mobilization in promoting gender-balanced participation and women’s potentially faster post-donation recovery (21). In terms of occupational distribution, corporate employees accounted for 34.33% and students for 26.85%, a pattern that was spatially consistent with the 54.49% representation of residents in newly developed residential communities, highlighting strong potential for blood donation behavior in urban “work-residence communities” during urbanization (22, 23). Additionally, respondents with bachelor’s degrees or above constituted 58.14%, further indicating a strong association between educational attainment and voluntary blood donation behavior: highly educated individuals not only better understand the technical safety of blood donation but also tend to show stronger social responsibility and self-efficacy, thereby enhancing willingness to participate (24–26) (correlation coefficient: 0.340). Research by Aschale et al., based on planned behavior theory, similarly confirms that education significantly enhances blood donation intention by strengthening perceptions of subjective norms and behavioral control (27).

From a practical perspective, this study identifies several key barriers to community-based platelet donation recruitment. Only 9.80% of respondents had prior blood donation experience, and among those with donation history, 59.18% donated only once in the past year, indicating insufficient sustained participation in the sample. At the cognitive level, 38.47% of respondents held misconceptions about the relationship between blood donation and health; 25.70% were unaware of the clinical beneficiaries of mechanically collected platelets; and 19.16% did not understand that component blood donation does not pose health risks. Regarding service accessibility, 31.34% of potential donors identified prolonged donation procedures as the primary barrier, 25.60% expressed concerns about blood-draw discomfort, and 19.21% worried about disruptions to work or daily routines. These findings are highly consistent with previous research on psychological barriers, information asymmetry, and time constraints affecting blood donation intention (28), underscoring that initiatives for platelet donation via machine collection should emphasize risk communication, process streamlining, and user-friendly service design. Such misconceptions may stem from excessive concerns about post-donation adverse reactions (29). Therefore, targeted education and awareness campaigns are essential for programs promoting platelet donation via machine collection (30). The effectiveness of health intervention measures depends not only on available resources but also on the organizational capacity of community engagement efforts (31).

At the recruitment level, multiple-response analysis indicates that short-form science communication has become the primary channel for information dissemination, with a penetration rate of 79.49% and a response rate of 26.44%, consistent with Yao et al.’s observation that “short videos have emerged as a key medium for health information dissemination” (32). Regarding community governance measures, the three core recruitment measures were “Party members taking the lead” (penetration rate: 71.71%), “free health check-ups and consultations” (penetration rate: 60.58%), and “special fund incentives” (response rate: 41.97%). The relatively high response rate for Party member-led initiatives (24.02%) not only reflects the traditional strength of political mobilization but may also reduce public psychological distance through empathetic communication (33).

Theoretically, the significance of this study lies in integrating social capital theory, community governance theory, and planned behavior theory to clarify how external community resources may influence individual blood donation behavior through attitudinal transformation. Organizational empowerment emphasizes trust networks and social support norms; cognitive empowerment highlights community philanthropic culture and transparency in blood usage; and service empowerment focuses on reducing participation barriers and facilitating the transition from attitudes to behaviors. The partial mediating effect of attitudes toward platelet donation identified in the structural equation modeling (SEM) results suggests that community-level trust, norms, and information disclosure may not directly “determine” blood donation behavior, but may partially shape behavioral tendencies by influencing residents’ value judgments, sense of security, and perceived feasibility of platelet donation for clinical use. This finding helps bridge macro-level community governance mechanisms and micro-level mechanisms of prosocial behavior.

This study further suggests that, within communities in China’s megacities, institutional trust, transparency in blood usage, and organized social support may serve as key contextual variables for understanding residents’ blood donation intention. However, because all participants were from Chongqing and the sample was predominantly composed of young adults, corporate employees, and students, the generalizability of these findings to other cities, rural areas, or different institutional-cultural contexts should be evaluated cautiously. The results also provide empirical support for Coleman’s classic social capital proposition that “cultural norms drive behavior.” Overall, the findings delineate a clear operational logic for the three-dimensional community empowerment model: at the organizational level, the tripartite network of “community party committees—grid-based branches—volunteer Party members” may transform political advantages into social exemplars; at the cognitive level, targeted health education may mitigate systematic bias; and at the service level, future research could assess whether VR technology alleviates anxiety among first-time donors or whether AI-powered appointment systems optimize procedures, although current cross-sectional data are insufficient to verify the actual efficacy of such digital interventions. Meanwhile, integrated resource coordination among the “community—enterprises—healthcare institutions” trio appears highly feasible: enterprises provide “public welfare hourly wages” to address 19.21% of work-related concerns, healthcare institutions dispatch teams to respond to 8.08% of health-related concerns, and community-specific funds incentivize regular blood donors through medical priority access, thereby preliminarily establishing a sustainable incentive mechanism.

This study still has several limitations. First, the cross-sectional design does not permit determination of temporal sequencing among variables and therefore cannot establish a causal effect of community empowerment measures on actual blood donation behavior. Second, because the data were primarily derived from self-reported questionnaires, the findings may be subject to social desirability bias, recall bias, and common method bias. Third, the analysis of blood donation behavior relied mainly on previous donor subgroups and behavioral tendency indicators, without longitudinal matching to actual donation records from blood banks. Fourth, the sample was limited to communities in Chongqing, whose megacity characteristics—including community governance structure and resident composition—may constrain the generalizability of the findings. Fifth, proposed recruitment measures such as short-video health education, government–enterprise collaboration, leadership by Party members, and process optimization remain candidate approaches inferred from survey results and require further validation through community intervention trials, longitudinal follow-up, and cost-effectiveness evaluation.

Based on a cross-sectional survey of community residents in Chongqing, this study developed and preliminarily validated a three-dimensional community empowerment model encompassing “organization, cognition, and service” for platelet donation recruitment in the context of platelet donation via machine collection. The results indicate that institutional trust, loyalty among regular blood donors, community philanthropic culture, transparency in blood usage, and social support norms all show statistically significant correlations with attitudes and behavioral tendencies toward platelet donation, with attitudes toward platelet donation serving as a mediating factor in specific pathways. This model provides a theoretical framework and potential recruitment measures for platelet donation recruitment in megacities; however, its direct intervention efficacy was not validated in this study. Future research should employ longitudinal studies, community intervention experiments, and cost-effectiveness analyses to further examine the model’s practical value.

## Data Availability

The original contributions presented in the study are included in the article/supplementary material, further inquiries can be directed to the corresponding authors.

## Appendix 1

**Table A1:** Measurement content and theoretical sources of questionnaire items.

Conception	Number of entries	Main measurement items	Theoretical correspondence
Institutional trust	6	Trust in blood banks, medical institutions, community organizations, and blood management processes	Organizational empowerment; Institutional trust; Social capital
Enhancing the loyalty of regular blood donors	8	Continuous donation willingness, intention to participate again, willingness to recommend others	Organizational empowerment; donor retention; social capital
Community public Welfare culture	8	The atmosphere of community public welfare, altruistic norms, and a sense of public responsibility	Cognitive empowerment; prosocial motivation; community culture
Blood transparency	7	Transparency of blood destination, information accessibility, risk communication, and feedback mechanisms	Cognitive empowerment; health beliefs; governance transparency
Social support norms	6	Family members, colleagues, community members, and Party officials set an example by providing support.	Organizational empowerment; subjective norms; social support
Attitudes toward donating platelets collected by machine	8	Evaluation of the safety, necessity, value, and participation convenience of automated platelet collection	Behavioral planning theory; attitude variable
Intention to donate blood	6	Previous blood donation history, frequency of blood donation, and future participation and recommendation tendencies	Behavioral outcome variable
